# Clinical Experience with Emicizumab and Rituximab as First-Line Treatment in a Case Series of Acquired Hemophilia A

**DOI:** 10.3390/hematolrep18020019

**Published:** 2026-03-05

**Authors:** Hikari Ota, Kyohei Yasuda, Namie Toyota, Kazuhiro Masuoka

**Affiliations:** Department of Hematology, Mishuku Hospital, Tokyo 153-0051, Japan; yasuda.1003.fesi@gmail.com (K.Y.); masuoka@mishuku.gr.jp (K.M.)

**Keywords:** acquired hemophilia A, emicizumab, rituximab

## Abstract

**Background:** Acquired hemophilia A (AHA) is a bleeding disorder caused by autoantibodies against coagulation factor VIII. Treatment includes controlling bleeding and eliminating the inhibitor. Emicizumab has been increasingly used to prevent bleeding in patients with AHA. Rituximab is used as a first-line immunosuppressive therapy (IST) for AHA, either in combination with corticosteroids in high-risk patients or as monotherapy in low-risk patients who cannot tolerate corticosteroids. However, evidence regarding concomitant emicizumab and rituximab as first-line treatment for AHA is limited. **Case presentations:** We present five cases of AHA diagnosed at a single institution. The first three high-risk AHA cases in the era before emicizumab resulted in poor outcomes due to bleeding (Cases 1 and 3) or infection (Case 2). The recent cases (Cases 4 and 5) were successfully treated with emicizumab and rituximab-containing IST without severe bleeding and infections. Since emicizumab effectively relieved pain in these patients, rehabilitation could be initiated promptly, resulting in earlier hospital discharge. Complete remission was achieved on Day 42 in Case 4 and on Day 22 in Case 5, respectively, and emicizumab was subsequently discontinued in both cases. **Conclusions:** Our case series suggests that early initiation of emicizumab for patients with AHA is effective in preventing severe bleeding and subsequent immobility, and it can be combined with rituximab-containing IST to achieve remission, potentially with fewer adverse effects than standard IST. Further studies are warranted to establish the optimal treatment protocol involving emicizumab and IST for AHA.

## 1. Introduction

Acquired hemophilia A (AHA) is a severe bleeding disorder caused by autoantibodies against coagulation factor VIII (FVIII). It carries a mortality rate of approximately 30%, with bleeding, infections related to immunosuppression therapy (IST), and underlying disease being major causes of death [1,2].

Treatment of AHA includes controlling bleeding and eliminating the inhibitor [3]. Emicizumab, a monoclonal anti-FIXa/FX bispecific antibody that substitutes the function of FVIII, has been increasingly and effectively used to prevent bleeding in patients with AHA [4]. It was approved in Japan in 2022 for this indication. Regarding IST for AHA, corticosteroid monotherapy is recommended for patients with FVIII > 1% and inhibitor titers ≤ 20 BU/mL, whereas combining corticosteroids with a cytotoxic agent (e.g., cyclophosphamide) or rituximab is preferred for patients with FVIII < 1% or titers > 20 BU/mL [3]. Rituximab can also be used as first-line IST for AHA when corticosteroids are not tolerated [3]. However, reports focusing on the concomitant use of emicizumab and rituximab as first-line treatment for patients with AHA remain scarce [5], and the optimal combination of emicizumab and IST has not been determined.

Herein, we present five cases of AHA where the latest two cases initially treated with emicizumab and rituximab illustrate successful clinical courses in contrast to the previous three cases without emicizumab. The concomitant use of emicizumab and rituximab was effective for preventing severe bleeding and subsequent immobility, and reducing corticosteroid-related adverse events, including infections.

## 2. Case Presentations

We identified five consecutive patients newly diagnosed with AHA at our hospital between April 2016 and March 2025. For each patient, we evaluated and analyzed age, sex, comorbidities, Eastern Cooperative Oncology Group Performance Status (ECOG PS) [6], bleeding sites before diagnosis, the presence or absence of severe bleeding, hemoglobin levels, FVIII activity levels, FVIII inhibitor titers, red blood cell transfusions, use of hemostatic agents, emicizumab use, the occurrence of bleeding after emicizumab administration, details of IST, infectious complications, occurrence of thrombosis, treatment response, and clinical outcomes. Severe bleeding was defined as follows: drop in hemoglobin of >2 g/dL, required > 2 packed red blood cell transfusions, and/or organ-, limb-, or life-threatening [7]. For patients receiving emicizumab, a loading dose of 6 mg/kg on Day 1 and 3 mg/kg on Day 2 was administered, followed by 1.5 mg/kg weekly as maintenance therapy from Day 8 onward [8]. Complete remission was defined differently depending on whether emicizumab was used. In patients not receiving emicizumab, it was defined as normalization of FVIII activity and eradication of FVIII inhibitors. In patients receiving emicizumab, FVIII activity was measured by a one-stage clotting assay using emicizumab-containing plasma samples neutralized ex vivo by adding two anti-emicizumab idiotype monoclonal antibodies (OSAwEN) [9]. Similarly, FVIII inhibitors were measured by a clotting time-based Bethesda assay with ex vivo emicizumab neutralization [9]. Complete remission was defined as FVIII activity levels > 50% measured by OSAwEN and the eradication of FVIII inhibitors. The OSAwEN method was performed up to 6 months after the final dose of emicizumab. For each case, the day of IST initiation was defined as Day 1.

**Figure 1:**
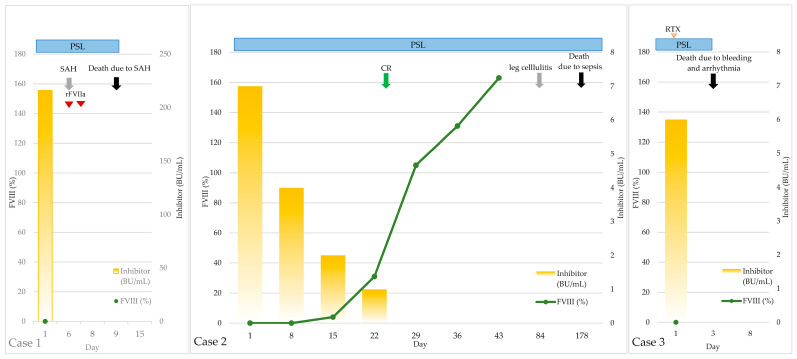

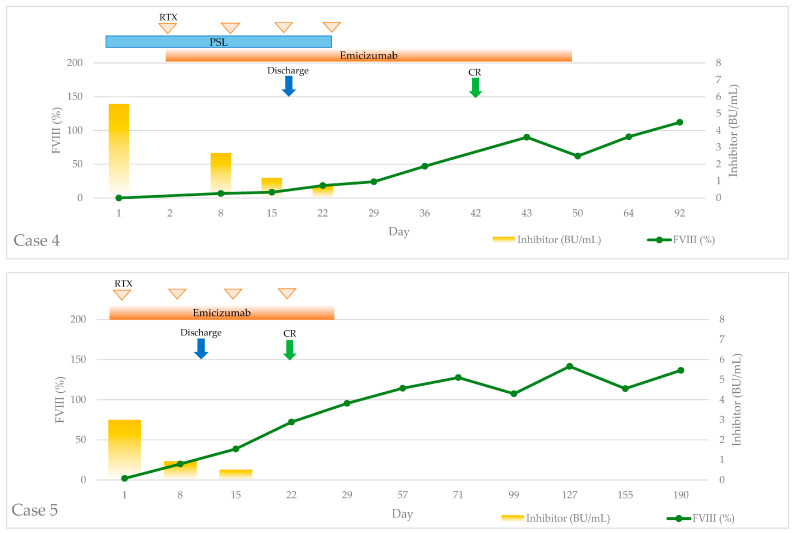
Clinical courses of the five patients. The timelines illustrate serial changes in FVIII activity and inhibitor levels in relation to therapeutic interventions and key clinical events. FVIII: coagulation factor VIII; BU: Bethesda unit; PSL: prednisolone; SAH: subarachnoid hemorrhage; rFVIIa: recombinant factor VII activated; RTX: rituximab; CR: complete remission.

All five patients had bleeding complications and were found to have prolonged activated partial thromboplastin time (APTT), leading to the identification of an FVIII inhibitor. All patients except for Case 5 had received red blood cell transfusions for bleeds before diagnosis. None of the patients had received hemostatic agents before the diagnosis of AHA. The characteristics and clinical courses of the five cases are summarized in Table 1 and Figure 1, respectively. Each clinical course is described in detail below:

[Case 1] A 91-year-old man. Comorbidities: atrial fibrillation and dementia. ECOG PS: 2. Bleeding site: upper extremities. FVIII activity level and FVIII inhibitor titer at diagnosis were <1% and 216 Bethesda unit (BU)/mL, respectively. At the time of AHA diagnosis, the bleeding had already resolved; therefore, a bypassing agent was not required. He was started on 1 mg/kg of prednisolone on Day 1. The addition of a cytotoxic agent to prednisolone was not pursued, considering the patient’s general condition and age. The course was complicated by a traumatic subarachnoid hemorrhage after a fall on Day 6. The administration of recombinant factor VII activated (rFVIIa) at a dose of 90 μg/kg every 3 h for two days was not effective, and he died from the bleeding complication on Day 9.

[Case 2] A 94-year-old man. Comorbidities: interstitial pneumonia. ECOG PS: 3. Bleeding site: right iliopsoas muscle. FVIII activity level and FVIII inhibitor titer at diagnosis were <1% and 7 BU/mL, respectively. At the time of AHA diagnosis, the bleeding had already resolved without the need for bypassing agents. He was started on 1 mg/kg of prednisolone on Day 1. He achieved complete remission on Day 24, and the high-dose prednisolone was tapered from Day 29 to 10 mg/day as a maintenance dosage for interstitial pneumonia. His poor ECOG-PS status remained unchanged due to prolonged bedridden conditions after diagnosis of AHA. He was complicated by left leg cellulitis and recurrent aspiration pneumonia, and died of sepsis on Day 178.

[Case 3] A 72-year-old man. Comorbidities: diabetes mellitus and bullous pemphigoid. ECOG PS: 3. Bleeding site: left thigh and left flank muscle. FVIII activity level and FVIII inhibitor titer at diagnosis were <1% and 6 BU/mL, respectively. At the time of AHA diagnosis, the bleeding was considered resolved, as the hemoglobin level remained stable. He was treated with 0.5 mg/kg of prednisolone and 375 mg/m^2^ of rituximab on Day 1. On the afternoon of Day 3, he suddenly died of ventricular fibrillation, possibly triggered by anemia due to acute hemorrhage from recurrent muscle bleeds suggested by blood tests performed during cardiopulmonary resuscitation, which revealed that his hemoglobin levels had decreased from 8.6 g/dL to 6.1 g/dL over 7 h. Hemostatic agents and red blood cell transfusions could not be administered because of rapid clinical deterioration. An autopsy was not performed.

[Case 4] An 81-year-old man. Comorbidities: diabetes mellitus type 2. ECOG PS: 3. Bleeding site: left thigh muscle. FVIII activity level and FVIII inhibitor titer at diagnosis were 3% and 7 BU/mL, respectively. He was started on 0.5 mg/kg of prednisolone on Day 1. Additionally, rituximab (375 mg/m^2^ weekly over four weeks) and emicizumab were initiated from Day 2 to spare steroid exposure and prevent severe bleeding. He was complicated by mild hematuria on Day 4, but hemostatic treatment was not needed. He could start rehabilitation from Day 5 because his left leg pain gradually disappeared after administration of emicizumab. He was discharged on Day 17. Prednisolone was tapered off and stopped on Day 23. He achieved complete remission on Day 42, and emicizumab was stopped thereafter. Follow-up of FVIII activity and inhibitor titers was limited to Day 92 because the patient was temporarily unable to attend the outpatient clinic for personal reasons. At 10 months after IST, he developed sepsis and severe hyperglycemia possibly attributable to poorly controlled diabetes, which were treated with antimicrobial agents and intensive insulin therapy. During this readmission period, he showed no bleeding diathesis and had a normal APTT. He has been in remission for 21 months after the diagnosis of AHA.

[Case 5] An 83-year-old woman. Comorbidities: diabetes mellitus type 2. ECOG PS: 2. Bleeding site: right elbow and ankle, and multiple finger joints. FVIII activity level and FVIII inhibitor titer at diagnosis were 2% and 3 BU/mL, respectively. Although she had not experienced severe bleeding, emicizumab was initiated on the day of AHA diagnosis (Day 0) to avoid unexpected and life-threatening bleeding events. Given the comorbidity of diabetes mellitus, rituximab monotherapy (375 mg/m^2^ weekly over four weeks) was initiated as IST on Day 1, instead of corticosteroids. She could start rehabilitation from Day 5 because her upper and lower limb pain resolved, and was discharged from the hospital on Day 9. She achieved complete remission on Day 22 without experiencing severe bleeding, and emicizumab was discontinued on Day 28. She has been in remission without infection or thrombosis for 9 months after diagnosis.

**Table 1:** Clinical characteristics and outcomes of five patients with acquired hemophilia A.

	Age/Sex	Comorbidities	ECOG PS	Bleeding Site Severity of Bleeding	FVIII Activity Inhibitor Titer (BU/mL)	Use of Emicizumab	IST	Major Complication After Treatment	Remission Status Time to Response	Outcome Follow-Up Duration
Case 1	91/F	Atrial fibrillation, Dementia	2	Upper limb Severe	<1% 216	No	PSL	Subarachnoid hemorrhage	N.A.	Death 9 days
Case 2	94/F	Interstitial pneumonia	3	Iliopsoas muscle Severe	<1% 7	No	PSL	Infection	CR 29 days	Death 178 days
Case 3	72/M	Diabetes, Bullous pemphigoid	3	Flank muscle Severe	<1% 6	No	PSL + RTX	Muscle bleeding + Arrhythmia	N.A.	Death 3 days
Case 4	81/M	Diabetes	3	Thigh muscle Severe	3% 7	Yes	PSL + RTX	Sepsis + Severe hyperglycemia	CR 42 days	Alive 21 months
Case 5	83/F	Diabetes	2	Joints of extremities Mild	2% 3	Yes	RTX	No	CR 22 days	Alive 9 months

BU: Bethesda unit; IST: immunosuppressive therapy; F: female; PSL: prednisolone; N.A.: not applicable; CR: complete remission; M: male; RTX: rituximab.

## 3. Discussion

We present five cases of AHA. The first three cases in the era without emicizumab resulted in poor outcomes due to bleeding (Cases 1 and 3) or infection (Case 2).Although different prognostic risk profiles should be considered, the recent cases (Cases 4 and 5) were successfully treated with concomitant emicizumab and rituximab without any severe bleeding, subsequent immobility, and corticosteroid-related adverse events.

Our cases highlight the potential benefit of prompt initiation of emicizumab in preventing severe bleeding and subsequent immobility, even in patients without a history of severe bleeding before diagnosis. Emicizumab has been widely used for bleeding prophylaxis in congenital hemophilia A with or without inhibitors [10,11]. The efficacy of emicizumab in AHA has also been shown in several studies [8,12,13]. From the perspective of ECOG-PS, although poor ECOG-PS is an unfavorable prognostic factor in AHA [2], early initiation of emicizumab has been reported to enable earlier initiation of rehabilitation to prevent immobility [14]. Based on this evidence, the Gesellschaft für Thrombose und Hämostaseforschung (GTH-AHA) working group recommended that bleeding prophylaxis with emicizumab is effective and should be considered at the time of diagnosis (100% consensus) [15]. On the other hand, emicizumab has not yet been fully incorporated into routine clinical practice. This is partly because emicizumab is currently approved only in Japan, and the guidelines were developed before its introduction [3]. Since patients with AHA exhibit considerable variation in terms of bleeding risk, bleeding site and severity, laboratory results, ECOG PS, and comorbidities, and given the potential risk of thrombosis and high cost of emicizumab, its use has been left to the discretion of individual clinicians. Here, our case series adds real-world experience to the existing literature regarding the management of AHA, both with and without emicizumab. As a background, according to the European Acquired Hemophilia Registry database (*n* = 501) [1], the majority of patients with AHA had bleeding episodes at presentation (94.6%); the bleeding event was reported as severe (70.3%), occurring spontaneously (77.4%) at subcutaneous (53.2%) and deep muscles or retroperitoneal bleeding (50.2%). These data imply the potential difficulty of detecting bleeds early for physicians and nurses treating AHA. As shown in our Cases 1 and 3, initial severe bleeds can directly result in poor outcomes; this report suggests the importance of prompt bleeding prophylaxis with emicizumab in patients with AHA, possibly irrespective of a history of severe bleeding events before diagnosis. Furthermore, this report is consistent with previous literature describing additional benefits of emicizumab use in AHA, including a reduced requirement for bypassing agents, shortened hospitalization, and a potentially lower risk of nosocomial infections [16,17,18]. On the other hand, emicizumab use carries several challenges, including the potential thrombotic risk associated with the concurrent use of bypassing agents, particularly activated prothrombin complex concentrate (aPCC) [10], the necessity for specialized laboratory testing [9,19], and the risk of development of anti-drug antibodies [20,21]. Recently, recombinant porcine FVIII (rpFVIII) has also become an established hemostatic option for acute bleeding in AHA [22]. Although its sequential use with emicizumab is increasingly adopted, clinical experience regarding rpFVIII use during emicizumab prophylaxis remains limited [23]. The clinical utility of emicizumab in AHA warrants further investigation in future studies, particularly focusing on prior bleeding severity, ECOG PS, thrombotic complications, and medical costs.

The combined use of rituximab with emicizumab was effective in achieving remission and reducing steroid-related adverse events in our cases. Generally, rituximab is well tolerated and can be considered as first-line therapy if standard IST is contraindicated, but may have limited efficacy if used as a single agent [3,24]; in a study of 12 patients treated with rituximab alone, seven achieved CR (58%), and the median time to CR was 106 days (range 25–184) [25]. These recommendations were established before the emergence of emicizumab, and recent studies have proposed that emicizumab can be used with reduced-intensity IST such as rituximab monotherapy or rituximab plus reduced corticosteroids [5,13]. The reported cases of AHA initially treated with concomitant emicizumab and rituximab are summarized in Table 2, where the combined use shows favorable outcomes [5,12,13,26,27,28], although the time to remission was delayed and variable. More recently, Poston et al. also showed excellent outcomes of 62 cases of AHA treated with emicizumab [23]. In that study, 41 of the 62 cases received rituximab-containing IST: rituximab monotherapy (*n* = 14), rituximab and corticosteroid (*n* = 10), and rituximab and other IST (*n* = 17). There were no fatal bleeding events after the initiation of emicizumab and no infections related to IST, although emicizumab was not initiated immediately after diagnosis in all cases, with a median time to initiation of 19 days (range: 0–1461). In the same study, CR was achieved in 9 of 14 patients (64%) treated with rituximab monotherapy and in 6 of 10 patients (60%) treated with rituximab and corticosteroids; however, data on the time to remission were not reported. Our cases (Cases 4 and 5) also demonstrated a favorable clinical outcome with emicizumab and rituximab-containing IST. Additionally, in this case series, rituximab-containing IST may be associated with a shorter time to remission than those of previously reported cases (Table 2). However, both the current two cases had low inhibitor titers, which should be considered when interpreting these findings. Nevertheless, these observations provide valuable data that will guide future research in this field to establish the optimal strategy including discontinuation of the high-cost emicizumab. This case series has several limitations, including its retrospective nature, small sample size, considerable heterogeneity in patient and disease characteristics, and the lack of a standardized IST regimen, particularly regarding corticosteroid doses and durations, and rituximab use. Prospective studies are warranted to determine the optimal IST in combination with emicizumab for patients with AHA.

In summary, we experienced five cases of AHA at a single institution, where the recent two cases initially treated with emicizumab and rituximab-containing IST had better outcomes without severe bleeding or corticosteroid-related adverse events. Our case series suggests that early initiation of emicizumab for patients with AHA may be a reasonable approach to reduce the risk of unpredictable, potentially fatal bleeding events and subsequent immobility, and it can be combined with rituximab-containing IST to achieve remission with fewer adverse effects than standard IST, with careful consideration of patients’ risk profiles. Further studies are needed to establish the optimal treatment protocol involving emicizumab and IST for AHA.

## Figures and Tables

**Table 2:** Characteristics of acquired hemophilia A cases initially treated with emicizumab and rituximab [5,12,13,26,27,28].

First Author and Year	Number of Patients	Age Sex	Severe Bleeding Before Emicizumab	Timing of Emicizumab Initiation	Severe Bleeding After Emicizumab	FVIII Activity Inhibitor Titer (BU/mL)	IST	IST-Related Infection	Remission Status Time to Response	Outcome
Knoebl, 2021 [13]	12	74 * (51–87) ^†^ M (*n* = 6) F (*n* = 6)	Yes (*n* = 8) No (*n* = 4)	3 * (1–13) ^†^ days afterinitial hemostatic therapy	No	<1% * (<1–1.1) ^†^ (<1–1.5) ^‡^ 22.3 * (3.5–2000) ^†^ (9–80) ^‡^	RTX (*n* = 2) RTX + PSL (*n* = 10)	No	CR (*n* = 12) 115 days * (67–185) ^†^	Alive (*n* = 12)
Hansenne, 2021 [26]	2	73 M	Yes	7 days after initial IST	No	<1% 15.2	RTX + mPSL + CSA	No	CR 75 days	Alive
		93 M	Yes	At diagnosis	No	1% 11	RTX + mPSL	No	CR 4 months	Alive
Yates, 2022 [27]	1	83 M	Yes	At diagnosis	Yes	<1% 197	RTX + PSL	No	PR 258 days	Alive
Happaerts, 2022 [28]	1	75 M	Yes	At diagnosis	No	<1% 135	RTX + mPSL	Yes	N.A.	Dead
Chen, 2023 [5]	11	77 * (47–93) ^†^ M (*n* = 5) F (*n* = 6)	Yes (*n* = 9) No (*n* = 2)	At diagnosis	Yes (*n* = 1) No (*n* = 10)	1% * (<1–9) ^†^ 33 * (9.2–107.5) ^†^	RTX (*n* = 8) RTX + PSL (*n* = 3)	No	CR (*n* = 8); PR (*n* = 2) 163 days * (63–267) ^†^; Refractory (*n* = 1)	Alive (*n* = 11)
Engelen, 2023 [12]	7	75 * (68–78) ^†^ M (*n* = 5) F (*n* = 2)	Yes (*n* = 6) No (*n* = 1)	3 * (0–5) ^†^ days after diagnosis	No	0% * (0–1) ^‡^ 112 * (31.9–190) ^‡^	RTX (*n* = 1) RTX + mPSL (*n* = 6)	Yes (*n* = 1) No (*n* = 6)	CR (*n* = 6) 153 days * (88–173) ^†^; N.A. (*n* = 1)	Alive (*n* = 6) Dead (*n* = 1)
Current Cases	2	81 M	Yes	At diagnosis	No	3% 7	RTX + PSL	No	CR 42 days	Alive
		83 F	No	At diagnosis	No	2% 3	RTX	No	CR 22 days	Alive

IST: immunosuppressive therapy; RTX: rituximab; PSL: prednisolone; CR: complete remission; mPSL: methylprednisolone; CSA: cyclosporine; PR: partial remission; N.A.: not applicable; *: median; ^†^: range; ^‡^: interquartile range (IQR).

## Data Availability

The data presented in this study are available on request from the corresponding author due to patient privacy.
